# The KISS1 Receptor as an *In Vivo* Microenvironment Imaging Biomarker of Multiple Myeloma Bone Disease

**DOI:** 10.1371/journal.pone.0155087

**Published:** 2016-05-09

**Authors:** Julia Dotterweich, Robert J. Tower, Andreas Brandl, Marc Müller, Lorenz C. Hofbauer, Andreas Beilhack, Regina Ebert, Claus C. Glüer, Sanjay Tiwari, Norbert Schütze, Franz Jakob

**Affiliations:** 1 Orthopedic Center for Musculoskeletal Research, Orthopedic Department, University of Würzburg, Würzburg, Germany; 2 Section Biomedical Imaging, MOIN CC, Department of Radiology and Neuroradiology, University Hospital Schleswig-Holstein, Campus Kiel, Kiel, Germany; 3 Interdisciplinary Center for Clinical Research Laboratory, Department of Internal Medicine II, University Hospital of Würzburg, Würzburg, Germany; 4 Department of Medicine III, Technische Universität Dresden Medical Center, Dresden, Germany; RWTH Aachen University Medical School, GERMANY

## Abstract

Multiple myeloma is one of the most common hematological diseases and is characterized by an aberrant proliferation of plasma cells within the bone marrow. As a result of crosstalk between cancer cells and the bone microenvironment, bone homeostasis is disrupted leading to osteolytic lesions and poor prognosis. Current diagnostic strategies for myeloma typically rely on detection of excess monoclonal immunoglobulins or light chains in the urine or serum. However, these strategies fail to localize the sites of malignancies. In this study we sought to identify novel biomarkers of myeloma bone disease which could target the malignant cells and/or the surrounding cells of the tumor microenvironment. From these studies, the KISS1 receptor (KISS1R), a G-protein-coupled receptor known to play a role in the regulation of endocrine functions, was identified as a target gene that was upregulated on mesenchymal stem cells (MSCs) and osteoprogenitor cells (OPCs) when co-cultured with myeloma cells. To determine the potential of this receptor as a biomarker, *in vitro* and *in vivo* studies were performed with the KISS1R ligand, kisspeptin, conjugated with a fluorescent dye. *In vitro* microscopy showed binding of fluorescently-labeled kisspeptin to both myeloma cells as well as MSCs under direct co-culture conditions. Next, conjugated kisspeptin was injected into immune-competent mice containing myeloma bone lesions. Tumor-burdened limbs showed increased peak fluorescence compared to contralateral controls. These data suggest the utility of the KISS1R as a novel biomarker for multiple myeloma, capable of targeting both tumor cells and host cells of the tumor microenvironment.

## Introduction

Multiple myeloma (MM) is one of the most common forms of hematological diseases, accounting for 10% of hematological cancers and 1% of all malignant tumors [1, 2]. Malignant plasma cells invade and proliferate within the bone marrow leading to a high occurrence of skeletal lesions. These malignant cell populations disrupt the normally tightly regulated process of coupled bone formation, mediated by osteoblasts, and bone resorption, mediated by osteoclasts. As a result, MM within the bone leads to the formation of osteolytic lesions resulting in hypercalcemia, bone pain, and pathological fractures decreasing the quality of life and survival of patients.

Skeletal lesions are the result of a tight interaction between, among others, MM and mesenchymal stem cells (MSCs) and other skeletal precursors of the bone marrow microenvironment, which deliver pro-survival signals and promote MM progression and chemo-resistance [3–7]. These signals are mediated by direct cell-cell contact via e.g. integrin receptors [8], by cytokines such as interleukin-6 (IL-6), hepatocyte, vascular and insulin-like growth factors and by transforming growth factor-beta, all derived from the bone marrow microenvironment. To maintain this microenvironment, MM cells restrict MSC or osteogenic precursor cell (OPC) differentiation to the osteogenic lineage [9], contributing to progression of myeloma bone disease and impairing bone regeneration potential. Because of the prominent role the bone marrow cells play in MM progression, identifying new molecules specific for the MM microenvironment would prove valuable for both diagnostic and therapeutic targeting.

GPR54, also known as the KISS1 receptor (KISS1R), is a G-protein-coupled receptor which, in conjunction with its ligand kisspeptin, stimulates phosphatidylinositol turnover and arachidonic acid release via activation of the mitogen-activated protein kinases and extracellular kinases 1/2 pathways [10]. Though primarily involved—via direct regulation of gonadotropin-releasing hormone from the hypothalamus—in the onset of puberty, sexual maturity, and pregnancy [11–13], kisspeptin has also been described as a tumor suppressor in melanoma metastasis [14], and more recently, in other tumor types [15–17]. Besides an autocrine mechanism, paracrine signaling between kisspeptin-expressing tumor cells and KISS1R-expressing stromal cells has also been suggested [15]. Therefore, the KISS1R and kisspeptin represent an intriguing signaling system which is of particular interest in MM where tumor-microenvironment interactions are pivotal to tumor progression.

Currently, diagnosis of MM relies on the detection of excessive monoclonal immunoglobulins in the blood and urine and the degree of bone marrow infiltration, though this technique is often insufficient to monitor disease progression [18] and fails to localize aberrant malignant plasma cell clones. Whole body radiography was previously the standard practice for site-specific assessment of MM bone disease. However, because this technique requires at least 30% bone loss prior to detection [19], patients frequently already suffer from severe skeletal involvement at the time of diagnosis. In recent years, more sensitive magnetic resonance imaging- or computed tomography-based techniques have been utilized to detect up to 80% more osteolytic lesions. These techniques, however, are expensive, complicated to perform, and yield mixed results depending on the location of the lesion [20]. In order to overcome these limitations, other sensitive, simple, cost-effective assays are needed to easily and conclusively identify MM bone lesions. Disease localization using advanced nuclear medicine imaging approaches may be suited if a specific and sensitive targeting molecule could be identified. Diagnostic methods that allow monitoring of early events in myeloma-affected bone lesions may provide information for individualized therapies and may offer a survival advantage, as treatments are currently only recommended for patients with active disease.

The aim of this study was to test whether KISS1R and kisspeptin are expressed in MM cells and cells of the tumor microenvironment, whether interactions between MM cells and skeletal precursors resulted in up-regulation of the KISS1R-kisspeptin system, and whether these changes in gene expression signature could be used as a tool to develop a novel biomarker for the MM microenvironment in myeloma bone disease suitable for diagnostics and therapy.

## Materials and Methods

### Primary cells and cell lines

Primary human MSCs were obtained from the cancellous bone from the acetabulum of patients that received a total hip arthroplasty. Cancellous bone was used in accordance with the local Ethics Committee (Medical Faculty of the University of Würzburg), which approved this study, and after receiving the written consent from the patients of the Orthopedic clinic. The isolation of MSCs was performed as previously described [21] and according to the following procedure: The cancellous bone was washed once with DMEM/Ham´s F-12 medium, 1:1 mixture (Life Technologies GmbH, Darmstadt, Germany). After centrifugation at 270 *g* for 5 min, the supernatant was removed and the cancellous bone was washed several times with DMEM/Ham´s F-12 medium, 1:1 mixture. The supernatant of each washing step was collected and the cells were pelleted by centrifugation (270 *g*, 5 min). The cell pellet was reconstituted in propagation medium, consisting of DMEM/Ham´s F-12 medium, 1:1 mixture, supplemented with 10% (v/v) heat inactivated FCS (Biochrom, Berlin, Germany), 50 μg/ml L-Ascorbic acid 2-phosphate (Sigma-Aldrich Chemie GmbH, Schnelldof, Germany), 100 U/ml penicillin (Life Technologies GmbH), and 100 μg/ml streptomycin (Life Technologies GmbH). Cells were plated at a density of 4x10^7^ vital cells/ml in 175 cm^2^ cell culture flasks (total volume: 25 ml) and were incubated for 48 h to 72 h at 37°C, 5% CO_2_, followed by washing with PBS. Adherent cells were expanded in propagation medium for 10 to 14 days in total to confluency. At confluency, MSCs were detached using 0.05% trypsin-EDTA (Life Technologies GmbH) and reseeded as required.

The human plasmacytoma cell line INA-6 [22] was incubated in RPMI 1640 complete medium consisting of RPMI medium 1640 (Life Technologies GmbH), 20% (v/v) heat inactivated FCS, 100 μg/ml gentamicin (Life Technologies GmbH), 2 mmol/l L-glutamine (Life Technologies GmbH), and 1 mmol/l sodium pyruvate (Sigma-Aldrich Chemie GmbH). In the absence of MSCs or OPCs, INA-6 cells were cultivated with 2 ng/ml recombinant human IL-6 (R&D Systems GmbH, Wiesbaden, Germany). The myeloma cell lines OPM-2 [23], MM.1S [24], AMO1 [25], and U266 [26] were grown in RPMI complete medium 1640 with 10% (v/v) heat inactivated FCS, 100 U/ml penicillin, 100 μg/ml streptomycin, 2 mmol/l L-glutamine, and 1 mmol/l sodium pyruvate. The BALB/c-derived MM cell line MOPC315.BM2-LucGFP [2, 27, 28] (hence forth referred to as MOPC) was cultivated in RPMI medium 1640, 20% (v/v) heat-inactivated FCS, 100 U/ml penicillin, 100 μg/ml streptomycin, 2 mmol/l L-glutamine, and 50 μmol/l beta-mercaptoethanol (suitable for cell culture) (Life Technologies GmbH). The human breast cancer cell line MCF-7 [29] was cultivated in DMEM high glucose medium (Life Technologies GmbH), 100 U/ml penicillin, 100 μg/ml streptomycin, 1 mmol/l sodium pyruvate, and 10 μg/ml insulin (Sigma-Aldrich Chemie GmbH).

### Osteogenic differentiation of MSCs

For generation of osteogenic precursor cells (OPCs), confluent MSCs were incubated for two weeks with DMEM high glucose medium supplemented with 10% (v/v) heat inactivated FCS, 100 U/ml penicillin, 100 μg/ml streptomycin, and differentiation additives (10 mmol/l beta-glycerophosphate, 100 nmol/l dexamethasone, and 50 μg/ml L-Ascorbic acid-2-phosphate (all from Sigma-Aldrich Chemie GmbH)). The respective control cells were cultivated in DMEM high glucose media. After 14 days, alkaline phosphatase activity and calcium deposits were stained using the Alkaline Phosphatase Leukocyte Kit 86-C (Sigma-Aldrich Chemie GmbH) and 1% (w/v) alizarin red S solution (Sigma-Aldrich Chemie GmbH), respectively, as described previously [30].

### Staining of INA-6 cells with CellTracker™ Green

Myeloma cells were stained with CellTracker™ Green 5-chloromethylfluorescein diacetate (CMFDA) (Lonza Group AG, Basel, Switzerland) according to manufacturer´s instructions. Briefly, 4.5x10^7^ cells were resuspended in 12.8 ml serum-free propagation medium containing 5 μmol/l CMFDA. After 15 min of incubation, cells were pelleted (270 *g*, 5 min) and resuspended in 16 ml RPMI complete medium. After another 30 min incubation, cells were washed once with PBS, resuspended at a cell density of 3x10^5^ cells per ml RPMI 1640 complete medium and incubated overnight. The next day, cells were prepared for co-culturing with MSCs and OPCs by washing them once with PBS and resuspending them in mixed media 1:1 (v/v), consisting of 1 part of MSC or OPC propagation medium and 1 part of RPMI complete medium.

### Preparation of RNA samples

A total of 5x10^4^ MSCs were seeded per cm^2^ in 6 well plates or 175 cm^2^ cell culture flasks and allowed to attach for one day. For experiments with OPCs, differentiation of MSCs followed as described above. One day before co-culturing, medium was replaced with mixed media 1:1 (v/v). Afterwards, 2x10^6^ (6 well plate) or 3.5x10^7^ (175 cm^2^ flask) CMFDA^+^ myeloma cells were added to MSCs or OPCs in a final density of 4x10^5^ cells/ml. As a control, respective MSCs or OPCs were incubated in the same volume of mixed media 1:1 (v/v). MSCs/OPCs were purified after 24 h of co-culture by fluorescence activated cell sorting (FACS) using a BD FACS Aria^TM^ III cell sorter (Becton Dickinson GmbH, Heidelberg, Germany) or by magnetic activated cell sorting (MACS) using CD45 MicroBeads (for INA-6 cells, Miltenyi Biotec GmbH, Bergisch Gladbach, Germany) or CD38 and CD138 MicroBeads (for all remaining myeloma cell lines). Purity of FACS- or MACS-sorted fractions was controlled by re-analysis of sorted fractions using the BD FACS Aria^TM^ III cell sorter or Axio Vert.A1 fluorescence microscope (GFP filter) (Carl Zeiss AG, Jena, Germany), respectively.

Indirect co-culture was performed for 24 h using a trans-well culture system (6 well format, 0.4 μm pore) (Corning, Inc., purchased from VWR International GmbH, Darmstadt, Germany) and equal cell densities as described above. As a control, MSCs or OPCs were incubated in mixed media, 1:1 (v/v).

For total mRNA preparation, cells were lysed in RA1 buffer (Machery-Nagel GmbH & Co. KG, Düren, Germany) supplemented with 1% beta-mercaptoethanol (AppliChem GmbH, Darmstadt, Germany) and stored at -80°C until use.

### mRNA isolation, cDNA synthesis, and PCR analysis

Total RNA isolation and purification was performed with the NucleoSpin^®^ RNA kit (Machery-Nagel GmbH & Co. KG) according to the manufacturer's instructions. cDNA of RNA samples was synthesized by using equal amount of total RNA (1 μg), M-MLV reverse transcriptase (200 U) (Promega GmbH, Mannheim, Germany), and random primers (1 μg) (Life Technologies GmbH) according to the manufacturer´s instructions. For semi-quantitative reverse transcriptase (RT) analyses, the following intron-spanning primers (designed using Primer3Plus software and obtained from Eurofins MWG Operon, Ebersberg, Germany) were used (5′-3′ forward and reverse, respectively): housekeeping gene *eukaryotic translation elongation factor 1 alpha 1 (EEF1A1)* (NM_001402.5), *CTGTATTGGATTGCCACACG* and *AGACCGTTCTTCCACCACTG*; *KISS1R* (NM_032551.4), *CTCTGACCGCCATGAGTGT* and *GCAGGTTGTACAGTGCGAAG*. Semi-quantitative RT-PCR was performed in a peqSTAR2x thermocycler (Peqlab Biotechnologie GmbH, Erlangen, Germany) in 30 μl assays comprising Green GoTaq^®^ Flexi Buffer (1x) (Promega GmbH), MgCl_2_ (1.7 mmol/l) (Promega GmbH), dNTPs (0.3 mmol/l) (Bioline, Luckenwalde, Germany), GoTaq^®^ DNA Polymerase (1 U) (Promega GmbH), cDNA (20 ng), and sequence-specific primers (each 5 pmol). PCR was run according the following thermocycler program: 95°C for 2 min, 95°C for 30 s, 54°C (*EEF1A1*) or 55°C (*KISS1R*) for 30 s, 72°C for 1 min, finished by 72°C for 5 min. PCR products (10 μl) were separated and detected using agarose gel electrophoresis and 0.5x GelRed^®^ Nucleic Acid Gel Stain (Genaxxon Bioscience GmbH, Ulm, Germany), respectively. PCR primers were checked for specificity using BigDye Terminator v3.1 cycle sequencing kit (Life Technologies GmbH), ABI 3130xL Genetic Analyzer (Applied Biosystems, Darmstadt, Germany), and the software BioEdit (Tom Hall, Ibis Therapeutics, Carlsbad, CA, USA) as well as by matching the sequences with the NCBI/Primer BLAST database.

### Western blot analysis

Preparation of whole cell lysates was performed as previously described [21]. Briefly, PBS washed cells were resuspended in 1x lysis buffer (Cell Signaling Technologies, Inc., Danvers, MA, USA) comprising complete, EDTA-free protease inhibitor (Roche, Mannheim, Germany), and PhosStop phosphatase inhibitor (Roche). After 10 min incubation on ice, cells were sonicated (Ultrasonic homogenizer, BANDELIN electronic GmbH & Co. KG, Berlin, Germany) followed by centrifugation (13700 *g*, 15 min, 4°C). After determining the protein concentration with the Roti^®^-Quant assay (Carl Roth, Karlsruhe, Germany), proteins were mixed with Laemmli buffer and boiled for 5 min at 95°C. Equal amounts of proteins (MSC and OPC lysates: each 50 μg protein; MM cell line lysates: each 30 μg protein; blocking experiment: each 30 μg protein) were loaded onto 15% sodium dodecyl sulfate polyacrylamide gels. After performing denaturing polyacrylamide gel electrophoresis, proteins were transferred by semi-dry blot onto polyvinyldifluoride membranes (WESTRAN^®^ clear signal; GE Healthcare Europe GmbH, Freiburg, Germany). Blocking of the membrane was performed for 1 h at room temperature in 5% (w/v) BSA (Sigma-Aldrich Chemie GmbH) / 0.1% TBST (for anti-KISS1 and anti-Actin antibodies) or 5% (w/v) skimmed milk powder (AppliChem GmbH) / 0.1% TBST solution (for anti-KISS1R antibody), followed by overnight incubation (4°C) with primary anti-KISS1 (1:500, rabbit, polyclonal, #ab19028, Abcam, Cambridge, United Kingdom), anti-KISS1R (1:1000, rabbit, polyclonal, #AKR001, Alomone Labs, Ltd., Jerusalem, Israel) or anti-Actin (1:1000, rabbit, monoclonal, #4970, New England Biolabs, Inc., Beverly, MA, USA) antibody. TBST-washed membranes were exposed for 2 h at room temperature to horseradish peroxidase-conjugated secondary antibody goat-anti rabbit IgG (whole molecule)-peroxidase (1:5000, goat, polyclonal, #A0545, Sigma-Aldrich Chemie GmbH), washed again and developed using the Western Bright^TM^ Sirius^TM^ kit (Advansta, purchased from Biozym Scientific GmbH, Hessisch Oldendorf, Germany) according to manufacturer´s instructions. Signals were recorded with FluorChem FC3 (Cell Biosciences, purchased from Biozym Scientific GmbH, Hessisch-Oldendorf, Germany) and software AlphaView, Version 3.2.2 (Cell Biosciences, Inc., Palo Alto, CA, USA). For blocking experiment, the KISS1R antibody was pre-incubated with the KISS1R antigen peptide (C)GSWHPRSYAAYALK (Alomone Labs) in a ratio of 1:4 for 30 min at room temperature.

### Alexa 633-kisspeptin probe conjugation, purification, and quality control

The 54 amino acid kisspeptin, modified to include an N-terminal cysteine residue for site-specific fluorescent labeling, was synthesized by Genecust (Dudelange, Luxembourg). 1 mg peptide was dissolved in 1.6 ml PBS (final concentration 100 μM) and treated with 10-fold molar excess of tris(2-carboxyethyl)phosphine (Life Technologies GmbH) for 3 min at room temperature. Reduced peptide was then incubated with 10-fold molar excess (1 mg dye in 830 μl PBS to generate a 1 mM working stock) of Alexa Fluor 633 C5 Maleimide (Life Technologies GmbH). Incubation was carried out at room temperature in the dark for 2 h. Conjugated peptide was purified twice using a Sephadex PD-10 column (GE Healthcare Europe GmbH). Assessment of conjugation efficacy was performed by reverse phase high performance liquid chromatography (HPLC) using a MultoHigh-Bio200-5uC18 column (CS Chromatographie Services GmbH, Langerwehe, Germany) in a Shimadzu prominence HPLC machine (Duisburg, Germany). Samples were diluted in water containing 0.1% trifluoroacetic acid (Carl Roth, Karlsruhe, Germany). Samples were measured at 640 ms intervals for 50 min.

### Fluorescence microscopy

MSCs were seeded in a Nunc^TM^ Lab-Tek^TM^ II 4-well chamber slide (6.7x10^3^ cells/well) (Thermo Fischer Scientific GmbH, Erlangen, Germany) and allowed to attach overnight. Afterwards, the medium was replaced by 500 μl mixed media, 1:1 (v/v), followed by 24 h incubation. The day after, 500 μl mixed media, 1:1 (v/v) containing 1x10^4^ INA-6 cells or 1.5x10^5^ MOPC cells, 500 μl MM cell-derived supernatant (conditioned media) or 500 μl mixed media, 1:1 (v/v) (control) was added per well. For preparation of the conditioned media, INA-6 cells (2x10^5^ cells/ml) or MOPC cells (3.75x10^4^ cells/ml) were cultured for one day in mixed media, 1:1 (v/v), followed by centrifugation (270 *g*, 5 min) and filtration of the supernatant (filter pore size: 0.45 μm). After 24 h, cells were incubated for 30 min with either free dye or the Alexa 633-kisspeptin (40 μmol/l) at room temperature. After rinsing three times with PBS, cells were fixed in 4% paraformaldehyde and mounted with Vectashield mounting media for fluorescence containing 4',6-diamidino-2-phenylindole (Vector Laboratories, Inc., purchased from Linaris, Dossenheim, Germany). Images were acquired using a BZ-9000 fluorescence microscope (Cy5 filter) and the softwares BZ-II Viewer and BZ-II Analyzer 2.1 (all from Keyence, Neu-Isenburg, Germany).

### Animal experiments

*In vivo* binding of Alexa 633-kisspeptin was tested in immune competent BALB/c and CD-1 nude mice, inoculated with MOPC cells and MCF-7 cells, respectively. All experiments were performed according to the German regulations for animal experimentation. The study was approved by the Regierung von Unterfranken as the responsible authority (Permit Number 55.2–2531.01-76/10 and -103/11) and by the institutional authorities of the Ethics Committee for Animal Experiments at the Christian Albrechts University of Kiel (V242-7224.121–17). 1x10^6^ MCF-7 cells transfected with the near-infrared fluorescent protein [31] were suspended in 5 μl PBS and injected directly into the right tibia, with equal amounts of PBS injected into contralateral control limbs, of 3 female CD-1 nude mice (Charles River). Tumor progression was monitored using the NightOwl planar imaging system (Berthold Technologies, Bad Wildbad, Germany). 1x10^5^ MOPC cells, suspended in 100 μl PBS, were injected *i*.*v*. via the lateral tail vein into immune-competent female BALB/c mice (Charles River). 19 days after the injection of the MM cells, mice were imaged by *in vivo* bioluminescence (BLI) to identify 6 mice which contain lesions in only one of the hind legs. All animals were kept in temperature and humidity-controlled, pathogen-free environment, with a 12 h light/dark cycle, and access to food and water *ad libitum*. All experiments were conducted before disease burdened resulted in reduced appetite, decreased body weight or increased sensitivity to touch.

### *In vivo* imaging

BLI was performed with an IVIS Spectrum (Caliper-Xenogen, Alameda, CA, USA) as previously described [32]. Briefly, mice were anesthetized i.p. with a mixture of ketamine (100 mg/kg) and xylazine (10 mg/kg) in PBS. Luciferin (150 mg/kg) was co-injected and BLI measurements were started 10 min later. For MCF-7 mice, animals were imaged every minute following injection of 100 μl of Alexa 633-kisspeptin (40 μM) using the NightOwl. For MM-containing mice, a fluorescence pre-scan (excitation filter: 605 nm, emission filter: 660 nm; illumination time: 1 s) was performed using the IVIS Spectrum. Next, mice were injected with 100 μl of Alexa 633-kisspeptin (40 μM) in the lateral tail vein and immediately imaged every two minutes for up to 20 min using the fluorescence settings as described above. In addition, fluorescent images were acquired after 30, 45, and 60 min. At the end of the experiment mice were then euthanized, organs were prepared, and fluorescence *ex vivo* imaging of the organs was performed. Imaging data was analyzed with Living Image 4.0 (Caliper-Xenogen, Alameda, CA, USA) and Prism 5 software (GraphPad, La Jolla, CA, USA).

### Statistical analyses

All statistical analyses were conducted using Prism 5 software. Comparison between groups was made using either a paired or unpaired two-sample *t*-tests. Comparison of organ fluorescence was conducted using a 1-way ANOVA and Turkey’s multiple comparison test. Alexa 633-kisspeptin binding kinetics were quantified using association then dissociation curves with time_0_ = 25 min and NS = 1. P values of <0.05 were considered to be statistically significant.

## Results

### The KISS1R is upregulated in MSCs and OPCs after direct co-culturing with MM cells

**Fig 1:**
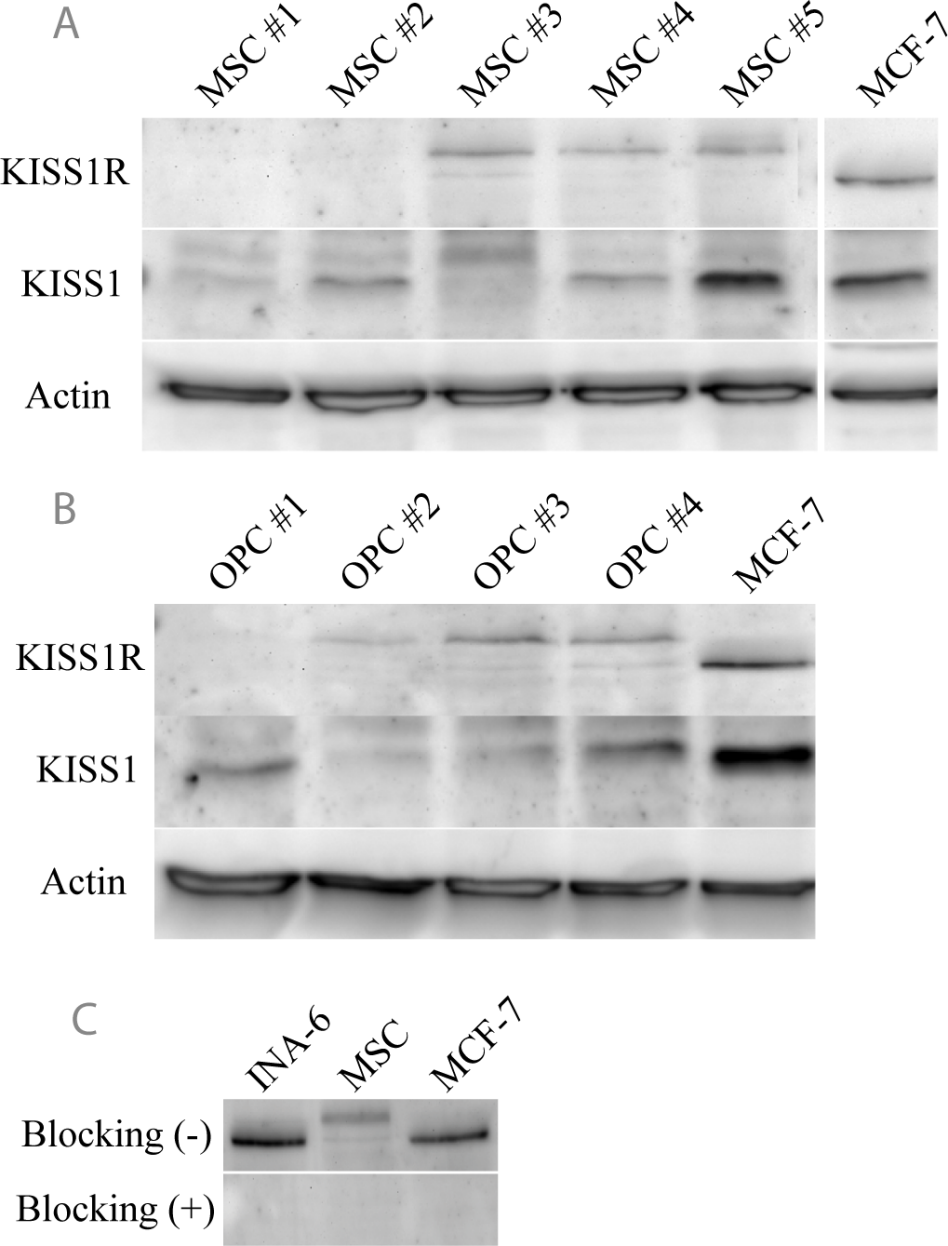
KISS1 and KISS1R are variably expressed in MSCs, OPCs, and tumor cell lines. Protein expression of the kisspeptin precursor KISS1 and the receptor KISS1R was investigated in primary MSCs (A) and OPCs (B). Specificity of the KISS1R antibody was confirmed by the addition of a blocking antigen (C). Actin was used as a loading control. Dividing lines indicate samples, which were loaded on different gels.

Protein levels of the KISS1R and the kisspeptin precursor KISS1 were analyzed by western blot in primary MSCs and MSC-derived OPCs from healthy donors (Fig 1A and 1B). MSCs and OPCs show low level, heterogeneous expression of both receptor (KISS1R) and ligand (KISS1). KISS1R expressed in skeletal precursor cells was also found to be of a greater molecular weight than that expressed in the MM cell line INA-6, as well as in the breast cancer cell line MCF-7 (Fig 1C) previously characterized to express high levels of the KISS1R [33]. Both are specific immunoreactive bands, since addition of blocking peptide attenuated antibody detection (Fig 1C). Therefore, this result may indicate presence of alternative splicing isoforms as has been previously described [34].

**Fig 2:**
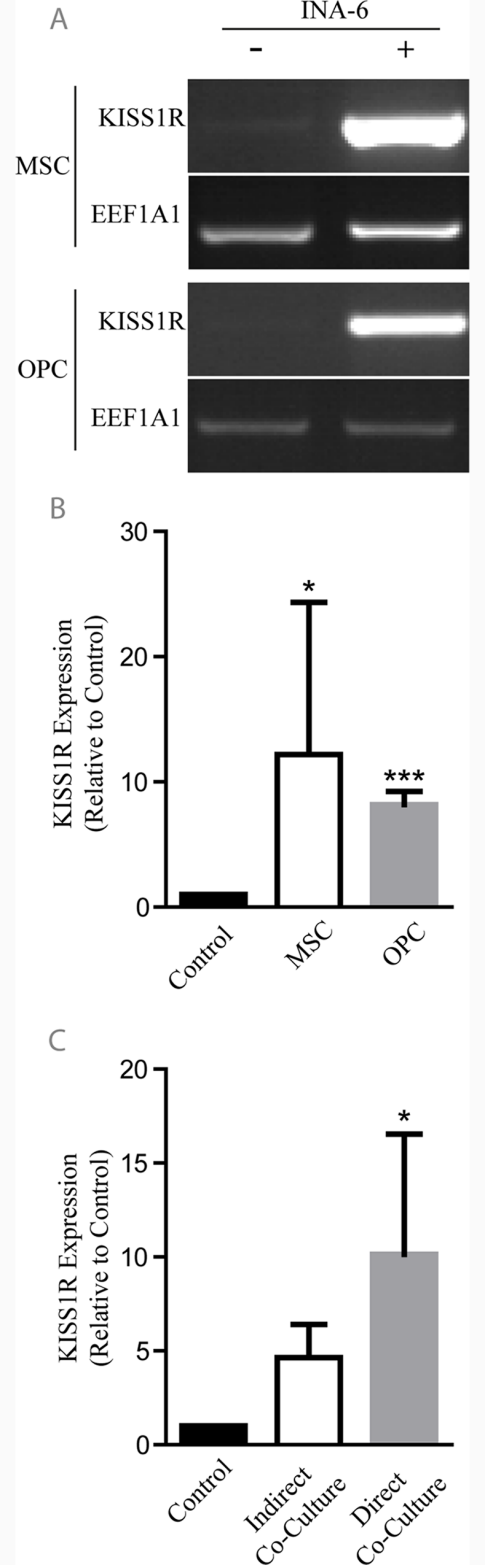
Direct co-culturing with INA-6 cells significantly upregulates *KISS1R* expression in MSCs and OPCs. *KISS1R* expression analyses of MSCs and OPCs after direct co-culturing with the MM cell line INA-6 (A). Both MSCs and OPCs express significantly higher *KISS1R* levels (relative to *EEF1A1* controls) in response to direct contact with MM cells (n = 5) (B). Up-regulation of *KISS1R* expression in MSCs is mainly contact-dependent, since indirect trans-well co-culturing techniques could not significantly elevate *KISS1R* expression (n = 3) (C). Graphs represent average values ± SD. (*p<0.05, ***p<0.001). Dividing lines indicate if samples were loaded on different gels.

To determine if changes in KISS1R expression can be expected following MM cell contact, *KISS1R* mRNA expression was assessed in MSCs and OPCs after direct or indirect culturing with INA-6 cells. Both MSCs and OPCs showed significantly higher mRNA expression after direct co-culturing with INA-6 cells (Fig 2A and 2B). This upregulation of *KISS1R* was found to be mediated only by direct INA-6 cell contact, since indirect co-culturing using a trans-well setup failed to show a significant increase in *KISS1R* mRNA of MSCs relative to controls (p = 0.1764) (Fig 2C). In addition, physical interaction with myeloma cell lines AMO1 and U266 clearly enhanced expression of *KISS1R* mRNA in bone-forming cells (S1 Fig).

### Fluorescently-labeled kisspeptin binds to MSCs *in vitro* after direct culturing with MM cells

**Fig 3:**
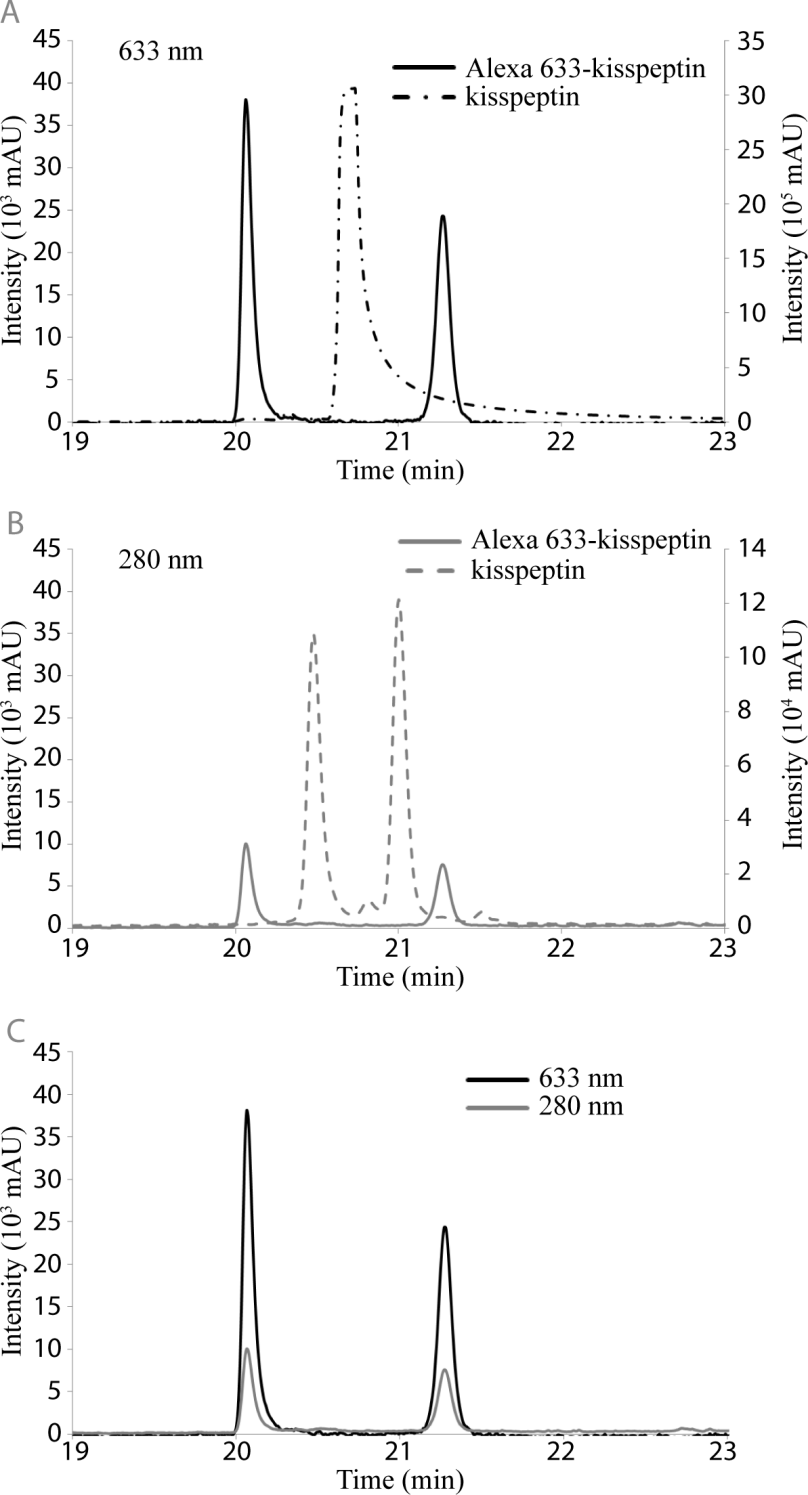
The fluorescent dye Alexa 633 successfully conjugates to the kisspeptin after maleimide-based reaction. HPLC confirmed the successful conjugation of kisspeptin with Alexa 633. Generation of the Alexa 633-kisspeptin probe results in a notable shift in elution time, measured at both 633 nm (A) and 280 nm (B) with no detectable unlabeled peptide. Elution peaks at both wavelengths are identical (C), indicating successful conjugation of the Alexa 633 dye and kisspeptin.

In order to detect upregulation of the KISS1R on MSCs after direct contact with MM cells, kisspeptin was synthesized with an additional N-terminal cysteine residue to permit labeling with the fluorescent dye Alexa Fluor 633 via a maleimide conjugation. Successful conjugation was confirmed by HPLC, which showed shifted peaks at both 633 nm and 280 nm for the conjugated peptide, relative to the unconjugated kisspeptin (Fig 3A and 3B). Shifted peaks were found to overlap, supporting a successful conjugation strategy (Fig 3C). The utility of Alexa 633-kisspeptin was tested first *in vitro* using cultured MCF-7 cells, and *in vivo*, using mice intratibially injected with MCF-7 cells (S2 Fig). *In vitro*, the conjugated kisspeptin probe bound to MCF-7 cells (S2A Fig), while *in vivo*, significantly elevated signals were observed in tumor-burdened tibiae relative to contralateral controls (S2E Fig).

**Fig 4:**
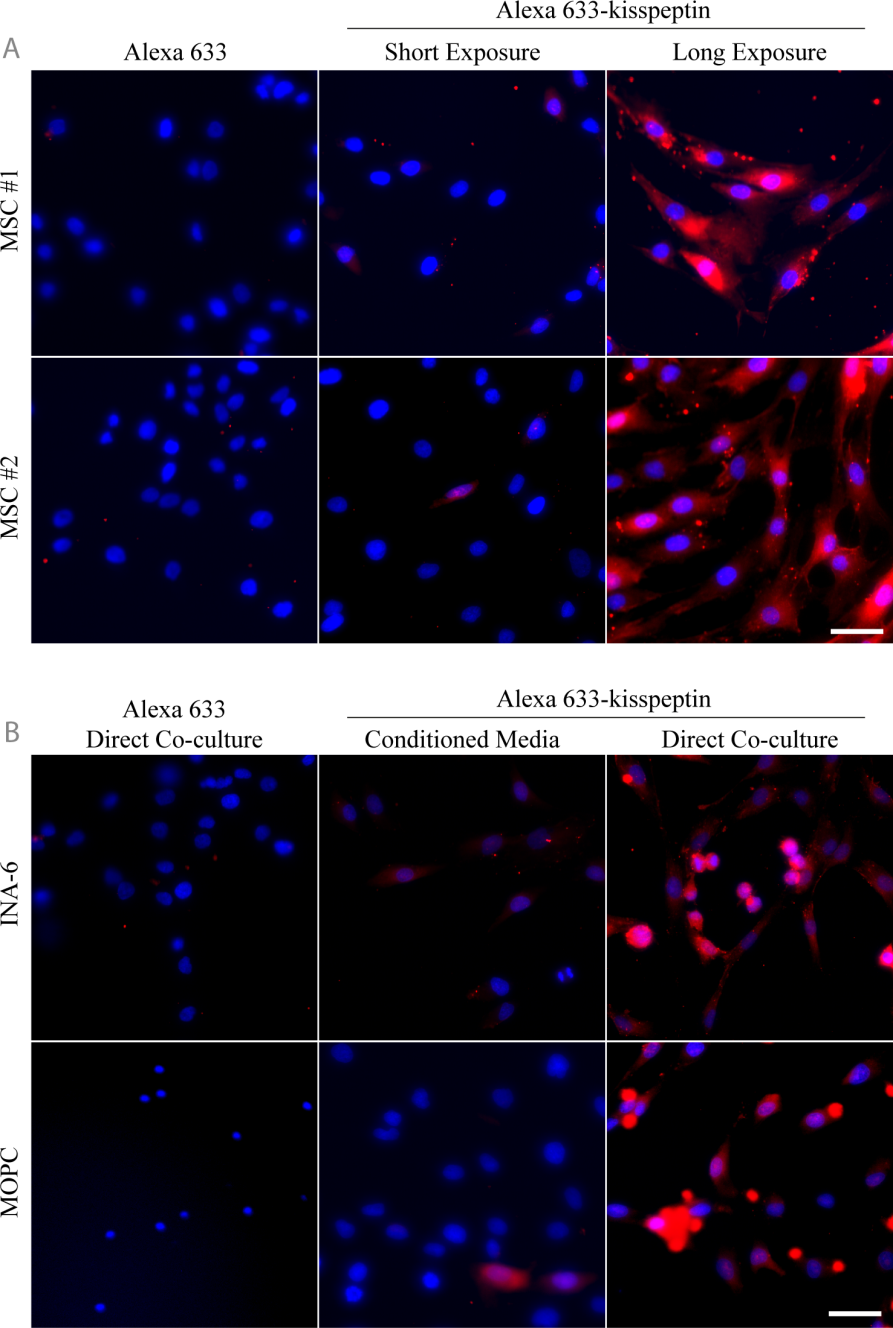
Alexa 633-kisspeptin binding to MSCs increases after direct co-culture with either human or mouse-derived MM cells. Primary MSCs from two different donors were incubated with Alexa 633-kisspeptin or the unconjugated dye and imaged with fluorescence microscopy (A). Dye shows heterogeneous, low level binding to MSCs in a kisspeptin-dependent manner. MSCs were incubated for 24 h with either MM cell line-derived media (conditioned media) or were directly co-cultured for 24 h with INA-6 cells or MOPC cells (B). Binding of the Alexa 633-kisspeptin probe required direct co-culture, while the free dye failed to show any specific binding. Bar = 50μm.

Primary MSCs were incubated with either free dye or the conjugated Alexa 633-kisspeptin and imaged by fluorescence microscopy (Fig 4). While addition of free dye revealed no notable fluorescence, addition of Alexa 633-kisspeptin showed low level, heterogeneous binding to MSCs (Fig 4A). Longer exposure images revealed that Alexa 633-kisspeptin does bind to cultured MSCs at basal levels. To determine if co-culturing leads to an up-regulation of KISS1R protein expression, MSCs were cultured either in conditioned media or directly with human (INA-6) or mouse-derived (MOPC) MM cells, incubated with Alexa 633-kisspeptin, and imaged by fluorescence microscopy (Fig 4B). While MSCs cultured in conditioned media showed low level probe binding, similar to levels observed under normal culture conditions, both MSCs and MM cells bound the fluorescent kisspeptin-probe at high levels after direct co-culture conditions, supporting mRNA expression data.

### Alexa 633-kisspeptin shows increased binding in limbs containing MM bone lesions

**Fig 5:**
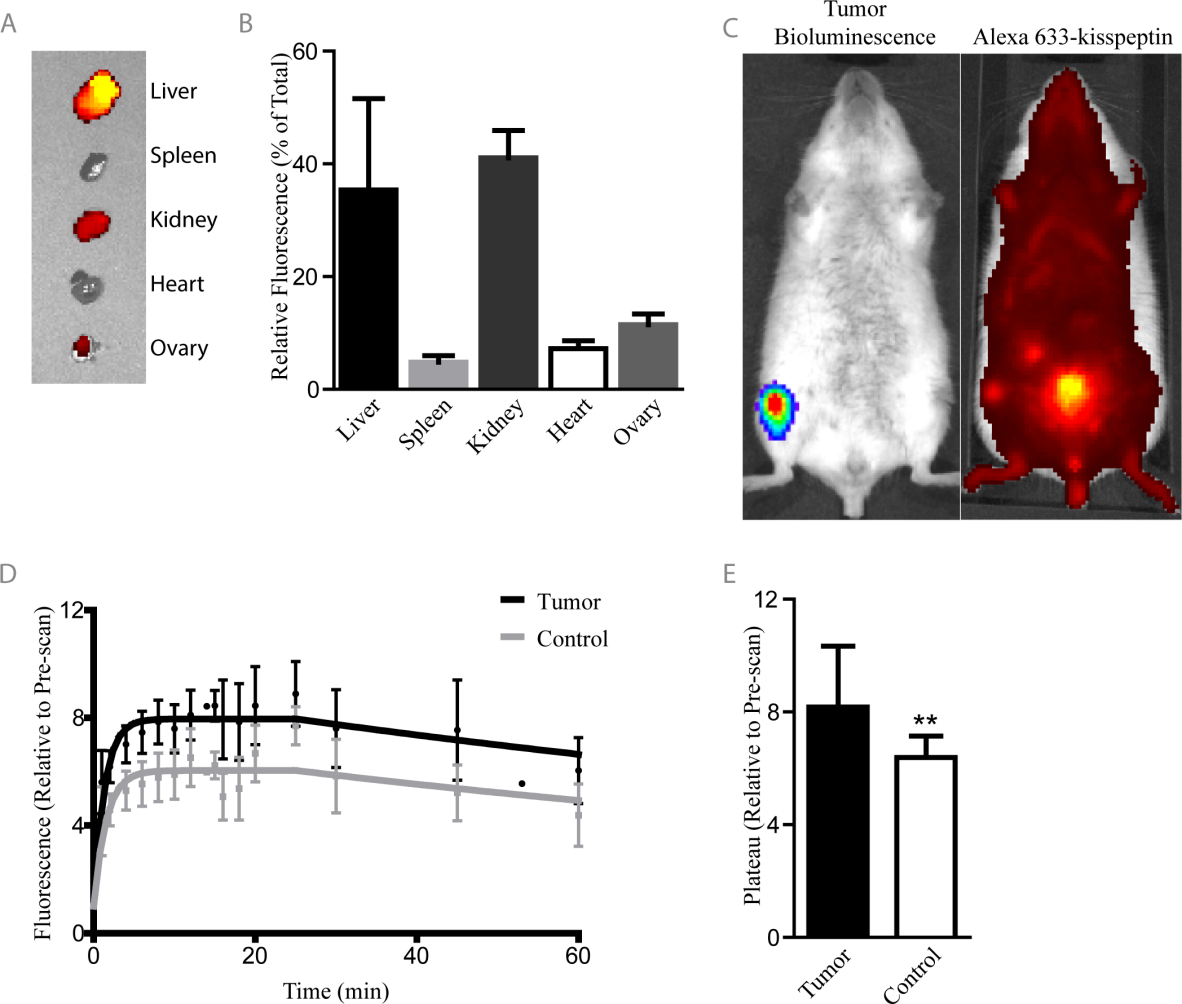
Alexa 633-kisspeptin binds to tumor-burdened limbs with increased peak binding and altered binding kinetics. Alexa 633-kisspeptin was injected into immune competent BALB/c mice and its biodistribution determined using the IVIS Spectrum (A-B). BALB/c mice intravenously-injected with the syngeneic MM cell line MOPC were monitored for tumor growth with bioluminescence imaging. Upon tumor development, mice were subsequently injected with Alexa 633-kisspeptin (C). Overall fluorescence of tumor-bearing limbs and their contralateral control limbs, relative to fluorescence detected in the pre-scan, were determined over time (D). Tumor-bearing limbs showed significantly increased relative peak fluorescence (E). Graphs represent average values ± SD. (n = 6) (**p<0.01).

To determine the biodistribution of the Alexa 633-kisspeptin and, importantly, to assess its feasibility as an *in vivo* biomarker for multiple myeloma, the kisspeptin probe was injected into naïve, immune-competent mice and organ uptake was quantified (Fig 5A and 5B). Along with the liver and kidneys, which showed significantly high levels of fluorescence (S1 Table), a notable amount of probe was also detected in the ovaries, an organ rarely associated with non-specific probe uptake and known to express high levels of KISS1R [35, 36]. To assess probe binding in the context of myeloma bone disease, we injected mice intravenously with the syngeneic firefly luciferase-expressing MM cell line MOPC and monitored sites of tumor formation with BLI (Fig 5C). Mice which had MM bone lesions in the proximal tibia/distal femur region of one leg only were injected with Alexa 633-kisspeptin *i*.*v*. and subsequently imaged for 1 h with time-lapse fluorescence imaging (Fig 5D). Significantly greater peak fluorescence levels were reached in tumor-burdened limbs as compared to contralateral sites (Fig 5E), indicating specific probe uptake at sites of MM manifestation.

## Discussion

Sensitive and early stage imaging of MM bone lesions is an important but challenging aim to improve active disease monitoring and to allow for timely therapeutic interventions in patients at the early onset of progressive disease. While traditional imaging techniques rely on the direct imaging of either the tumor itself or indirectly monitoring the resulting bone loss, an imaging biomarker sensitive to both the tumor and its surrounding microenvironment may provide a highly responsive tool in the diagnosis of MM bone lesions at early stages. In this study, we have identified the KISS1R as a novel biomarker for multiple myeloma bone disease.

KISS1R and KISS1 expressions varied in MSC and OPC from different donors, which may be explained by the intrinsic heterogeneity of MSC population. However, levels of *KISS1R* increased significantly in MSCs and OPCs when cultured with INA-6 cells, with the strongest effects under direct co-culture conditions. We confirmed these *in vitro* expression analyses with further experiments utilizing a fluorescently-labeled kisspeptin probe. Because of its higher stability [37, 38], the 54 amino acid form of kisspeptin was selected, though 10, 13, and 14 amino acid isoforms have also been identified as KISS1R ligands [39, 40]. Initial *in vitro* and *in vivo* screens using the KISS1R-expressing breast cancer cell line MCF-7 [33] revealed strong binding of the kisspeptin probe, validating its potential as a KISS1R marker. The Alexa 633-kisspeptin probe bound to MSCs only at low levels when cultured alone or with conditioned media derived from MM cells, but specific binding to both MSCs and MM cells increased markedly in a direct co-culture setting. We next sought to take advantage of this increased kisspeptin binding to MM and its microenvironment *in vivo*. A limited biodistribution analysis revealed uptake by the liver and kidneys, as is commonly observed for intravenously administered probes. Notably, fluorescent signal was also observed in the ovaries, an organ known to express high levels of the KISS1R and not traditionally associated with intravenously-injected probes [35, 36]. In the context of progressive multiple myeloma bone disease, probe detection increased significantly in tumor-burdened limbs when compared to the contralateral MM-free sites. These data provide strong evidence that the KISS1R is highly expressed within tumor-burdened regions of the skeleton and that this KISS1R upregulation can be utilized as an efficient biomarker of myeloma bone disease. Because the KISS1R is expressed not only by the MM cells themselves, but also by the surrounding stroma cells upon MM cell contact, this new biomarker has the potential to serve both as an early and sensitive diagnostic tool. Concomitantly, kisspeptin can be developed as a potent therapeutic targeting agent, which could help deliver therapeutic agents not just to the resistant tumor cells, but also to the cells forming the microenvironment, which should be significantly more sensitive to treatment and would in turn make MM cells more responsive to therapeutic interventions.

The data presented herein support KISS1R as a novel and promising new biomarker for myeloma bone disease, but still several questions remain. Firstly, while we have described the up-regulation of the KISS1R cells directly interacting with MM cells, little is known so far about the role of kisspeptin and the KISS1R in MM disease progression. It will be of great importance to understand the role of kisspeptin and the KISS1R in both bone marrow cells and MM, and to be aware of what potential side effects, either positive or negative, may be associated with targeting or activation of this system. While KISS1 is noted as a tumor suppressor in melanoma metastasis [14], the role of KISS1R signaling on breast cancer invasion is dependent on the estrogen receptor status of the tumor cells [41]. Secondly, the signal to background ratio obtained in this study could potentially be improved by enhancing the short half-life of kisspeptin. Since kisspeptin is cleaved and inactivated by matrix metalloproteinase 2 [42], an enzyme that plays a role in MM progression [43], imaging may benefit from the use of a non-cleavable form of the kisspeptin. Furthermore, rational modifications of kisspeptin have been described which could further increase the stability of the peptide [44–47].

In conclusion, the KISS1R provides a promising new opportunity for the diagnosis of MM bone disease. As a novel strategy it allows for targeting both the tumor cells and the host response to tumor arrival. This brings about an enhanced potential for monitoring especially early changes in bone microenvironment along disease development and also for treatment response.

## Supporting Information

S1 Fig Myeloma cell contact induces expression of *KISS1R* mRNA in MSCs and OPCs. MSCs and OPCs were co-cultured with CMFDA^+^ myeloma cell lines AMO1, MM.1S, OPM-2 or U266 for 24h, followed by separation using CD38 and CD138 MicroBeads. Representative image of agarose gels showing expression of *KISS1R* and *EEF1A1* (housekeeping gene) of respective MSC and OPC samples.
(TIF)

S2 Fig Alexa 633-kisspeptin binds to the KISS1R-expressing cell line MCF-7 *in vitro* and *in vivo*. Cultured MCF-7 cells were incubated with either free dye or dye conjugated to the modified kisspeptin and imaged with fluorescence microscopy (A). Immune-compromised mice were injected intratibially with MCF-7 cells and imaged for tumor formation using the NightOwl planar imaging system (B). Tumor-burdened mice were injected with Alexa 633-kisspeptin and imaged regularly for 20 min (C). Arrows point to tumor-burdened limbs. Tibia fluorescence was quantified over time (D) and plateau fluorescent values determined (E). Tumor-burdened limbs show significantly more probe uptake than the contralateral tumor-free limbs. Graphs represent average values ± SD. (n = 3) (*p<0.05).
(TIF)

S1 Table Statistical analyses of Alexa 633-kisspeptin probe biodistribution in BALB/c mice. Repeated measures ANOVA and Turkey’s multiple comparison test for organ accumulation of the Alexa 633-kisspeptin probe.
(DOCX)
