# Supplementary figures and images for: The KISS1 Receptor as an In Vivo Microenvironment Imaging Biomarker of Multiple Myeloma Bone Disease
Source: PLoS One. 2016 May 9;11(5):e0155087. doi: 10.1371/journal.pone.0155087 (PMC4861277; doi:10.1371/journal.pone.0155087)

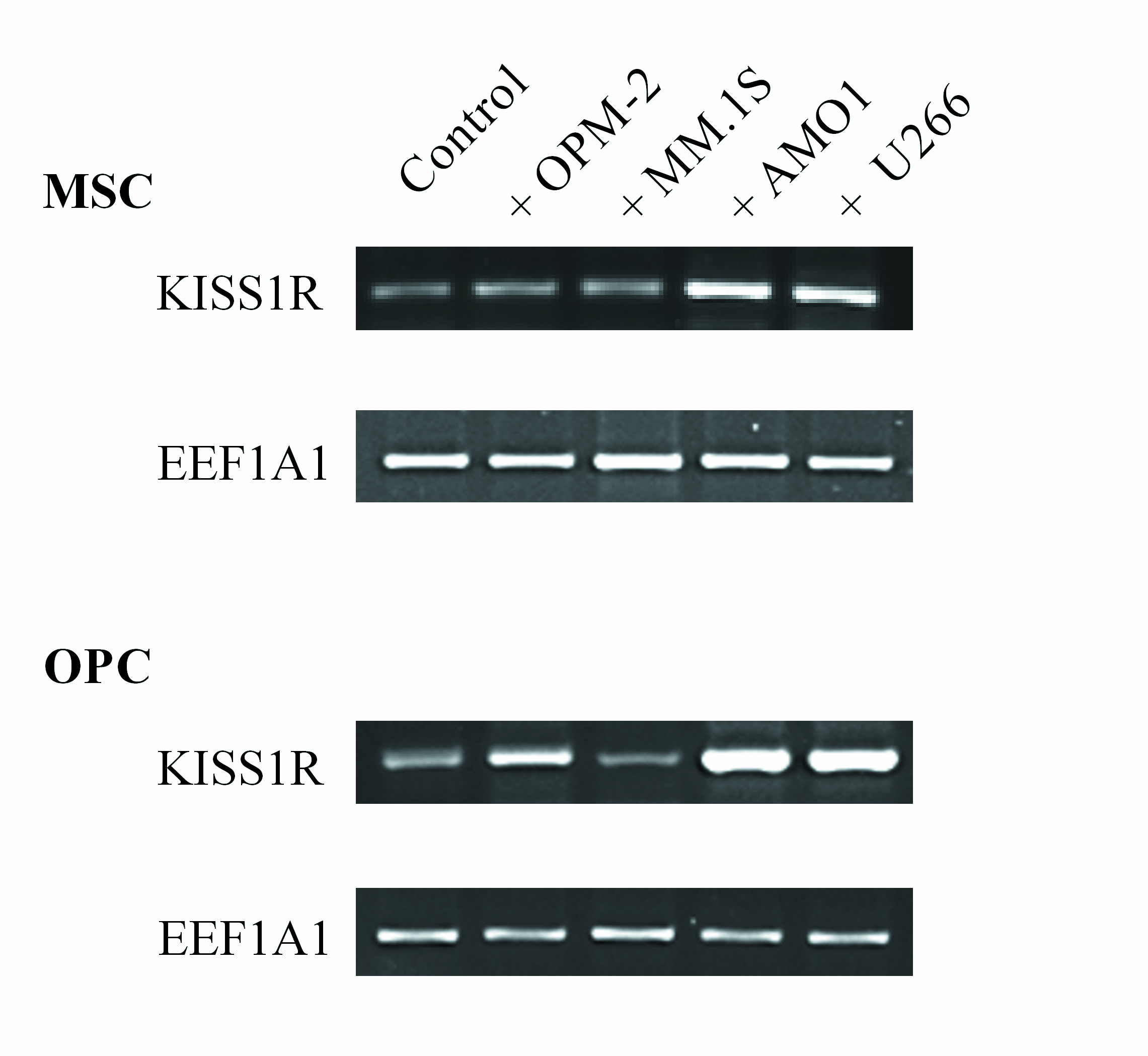

Supplement: S1 Fig — MSCs and OPCs were co-cultured with CMFDA+ myeloma cell lines AMO1, MM.1S, OPM-2 or U266 for 24h, followed by separation using CD38 and CD138 MicroBeads. Representative image of agarose gels showing expression of KISS1R and EEF1A1 (housekeeping gene) of respective MSC and OPC samples. (TIF) [file pone.0155087.s001.tif]

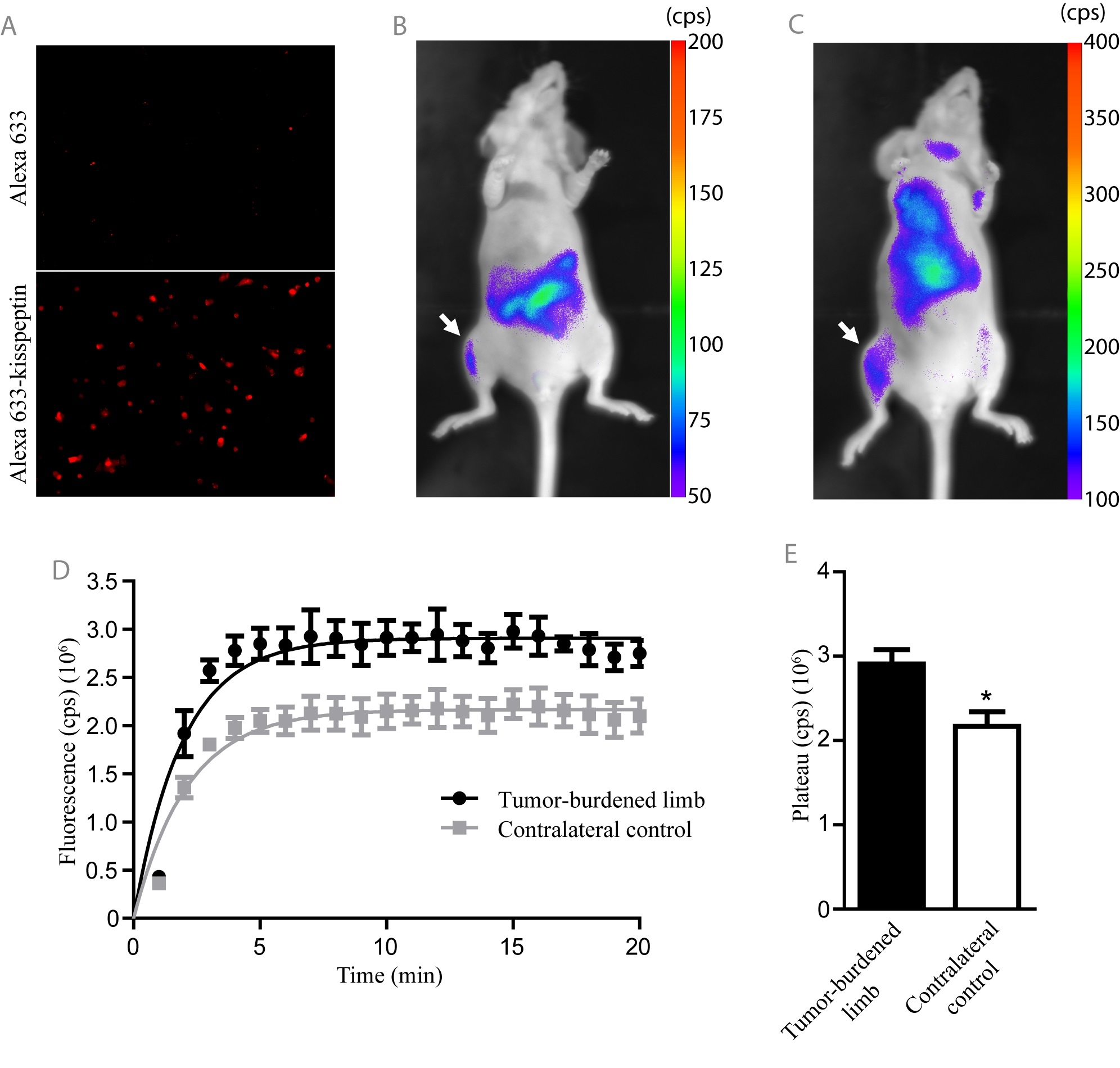

Supplement: S2 Fig — Cultured MCF-7 cells were incubated with either free dye or dye conjugated to the modified kisspeptin and imaged with fluorescence microscopy (A). Immune-compromised mice were injected intratibially with MCF-7 cells and imaged for tumor formation using the NightOwl planar imaging system (B). Tumor-burdened mice were injected with Alexa 633-kisspeptin and imaged regularly for 20 min (C). Arrows point to tumor-burdened limbs. Tibia fluorescence was quantified over time (D) and plateau fluorescent values determined (E). Tumor-burdened limbs show significantly more probe uptake than the contralateral tumor-free limbs. Graphs represent average values ± SD. (n = 3) (*p<0.05). (TIF) [file pone.0155087.s002.tif]
