# Supplementary material for: The KISS1 Receptor as an In Vivo Microenvironment Imaging Biomarker of Multiple Myeloma Bone Disease
Source: PLoS One. 2016 May 9;11(5):e0155087. doi: 10.1371/journal.pone.0155087 (PMC4861277; doi:10.1371/journal.pone.0155087)
Supplement: S1 Table — Repeated measures ANOVA and Turkey’s multiple comparison test for organ accumulation of the Alexa 633-kisspeptin probe. (DOCX) [file pone.0155087.s003.docx]

|  | Liver | Spleen | Kidney | Heart | Ovary |
| --- | --- | --- | --- | --- | --- |
| Liver |  | p<0.01 | ns | p<0.01 | p<0.05 |
| Spleen |  |  | p<0.001 | ns | ns |
| Kidney |  |  |  | p<0.001 | p<0.01 |
| Heart |  |  |  |  | ns |
| Ovary |  |  |  |  |  |
